# Retinal Racemose Hemangioma With Optic Neuropathy in a Child

**DOI:** 10.7759/cureus.17620

**Published:** 2021-08-31

**Authors:** Nur Nadia Ahmad Tarmizi, Mae-Lynn Catherine Bastion, Roslin Azni Abdul Aziz, Norshamsiah Md Din, Safinaz Mohd Khialdin

**Affiliations:** 1 Ophthalmology, Pusat Perubatan Universiti Kebangsaan Malaysia, Kuala Lumpur, MYS; 2 Ophthalmology, Universiti Kebangsaan Malaysia Medical Centre, Kuala Lumpur, MYS; 3 Ophthalmology, Hospital Universiti Kebangsaan Malaysia, Kuala Lumpur, MYS; 4 Ophthalmology, Hospital Shah Alam, Shah Alam, MYS

**Keywords:** ocular oncology & medical retina, oct angiography, arterio venous malformations, optic nerve atrophy, retina racemose hemangioma

## Abstract

The aim of this study is to report an unusual case of retinal racemose haemangioma (RRH) in a child resulting in optic neuropathy and its optical coherence tomography angiography (OCT-A) findings. This is a retrospective case report.

For almost a year, a 13-year-old girl experienced gradual, painless, generalized blurred vision in her right eye. Visual acuity was 6/60 with a positive relative afferent pupillary defect (RAPD) in her right eye. The right-eye fundus showed enlarged and tortuous retinal vessels extending from the optic disc to all four quadrants, including the juxta foveal region. OCT analysis revealed distortion in the region of enlarged vessels with minimal retinal fluid while OCT-A of the macula area demonstrated dilated and tortuous vessels in the superficial layers of the retina. Right intra-orbital vascular channels surrounding the optic nerve with optic nerve atrophy and gliosis were detected on magnetic resonance imaging angiography/venography (MRA/MRV). The cerebral angiogram reported an abnormal tangle of small vessels within the right orbit that received supply from a dilated right ophthalmic artery indicating the presence of retro-orbital arteriovenous malformation (AVM). She was then referred to the neurosurgeon and a decision was made not to embolize or resect the dilated vessel as this might lead to occlusion of the ophthalmic artery and thus worsen her vision.

RRH may present in the paediatric age group, and optic nerve atrophy is one of the disease manifestations. OCT-A is a less invasive diagnostic option compared to fundus fluorescein angiography (FFA) for diagnosis and monitoring of disease progression.

## Introduction

Arteriovenous malformation (AVM) of the retina or retinal racemose haemangioma (RRH) is a benign and rare developmental anomaly. It may occur as a solitary isolated lesion or as an integral part of Wyburn-Mason syndrome. The hallmark of RRH is a communication between the arterial and adjacent venous circulation. The severity of the ophthalmic presentation may vary, from a singular well-defined anastomosis limited to one quadrant of the fundus to an enormous tumour-like mass of dilated arteries and veins [1]. Such malformation in children is rarely observed [2]. Patients with isolated RRH are typically asymptomatic unless there are other associated problems such as venous occlusion, ocular ischaemia, and secondary glaucoma, which may cause visual impairment [2]. RRH causing optic neuropathy has seldom been reported. However, the presence of retinal AVM warrants brain and orbital imaging to rule out cerebral AVM [2]. Treatment of these malformed vessels includes embolization, sclerotherapy, surgical excision, or a combination of these within a multidisciplinary setting and specific treatment of its complications [2]. New imaging techniques, such as OCT-A, may aid in diagnosing and distinguishing the disease from other vascular pathologies.

## Case presentation

A 13-year-old girl was referred to our centre from a private ophthalmologist clinic for right eye retinal AVM. She first went to the private clinic with a complaint of progressive, painless, generalized blurring of vision for about one year, which was not worsening, and was elicited upon questioning. The parents noticed that she was straining her eyes when watching television. She also reported intermittent headaches, without nausea, vomiting, limb numbness, or weakness. The patient had no known medical illnesses, and she was born term with no significant perinatal and family history. Her psychomotor development was normal and she attended a regular school with average achievement.

She was prescribed glasses of −6.0D in both eyes by an optician two months before the private eye clinic visit. However, despite wearing glasses, her right eye vision remained poor. Hence, her parents decided to take her to an ophthalmologist.

On examination at our centre, the head and neck examination was normal and both eyes were symmetrically placed, with no proptosis or palpable pulsation. There were no facial naevi and the neurological examination done by paediatrician was normal.

Ocular examination revealed best-corrected visual acuity of 6/60, and unable to read for near in her right eye and 6/9, N6 in her left eye. Colour vision test using Farnsworth Dichotomous test series D-15 was normal in both eyes. The right relative afferent pupillary defect (RAPD) was positive. Intraocular pressure measured by Goldman applanation tonometry was equal between the two eyes and within normal limits. The ocular mobility and convergence were within normal limits and no phoria or strabismus was evident. Anterior segment examination was unremarkable with equal pupil size and no rubeosis.

**Figure 1:**
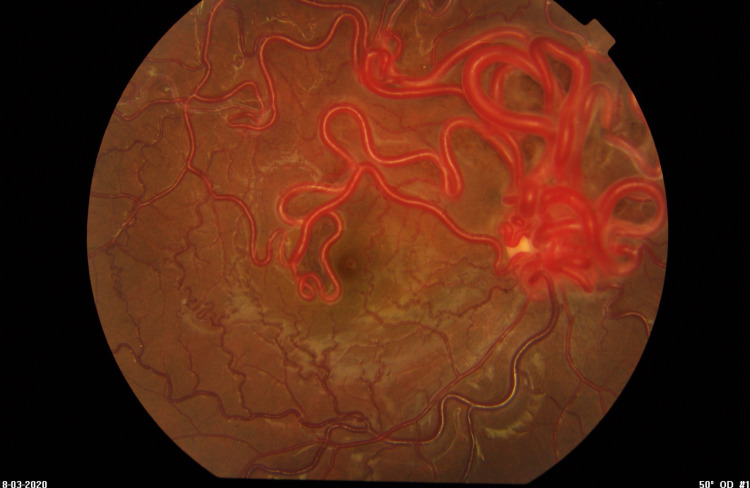
Fundus of the right eye showing massive and extensive dilated retinal vessels with severe tortuosity. Vessels are more prominent at the superior hemifield. Dilated vessels are obscuring the disc.

Dilated fundus examination of the right eye showed massive dilated retinal vessels in all four quadrants with severe tortuosity (Figure 1).

Numerous anastomosing vessels made the separation of arterial and venous components difficult. A more prominent vessel at the superior hemifield in a juxta foveal and temporal location of the macula was observed. These dilated vessels obscured the optic disc. Vessel dilatation extended from the juxta fovea location to the peripheral retina and was associated with tortuosity. Some sclerosed vessels were also seen at the periphery of the superior retina and macula. There was no exudation, haemorrhage, or abnormal pigmentation; and central macula reflection was normal. The left eye fundus showed a normal optic disc with no disc swelling, a cup to disc ratio of 0.3, and good foveal reflex with normal-looking retinal vasculature.

**Figure 2:**
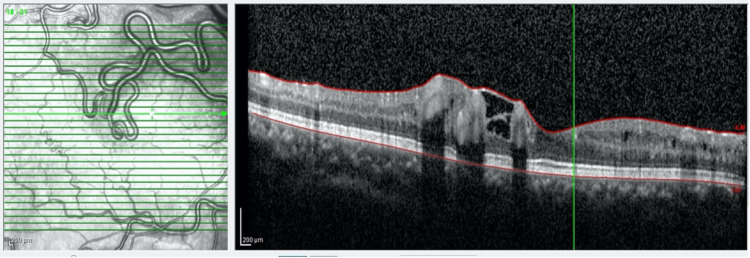
SD-OCT (SPECTRALIS®) macula examination showed the presence of minimal retinal fluid with distortion in the area of the enlarged vessels. SD-OCT: spectral-domain optical coherence tomography.

**Figure 3:**
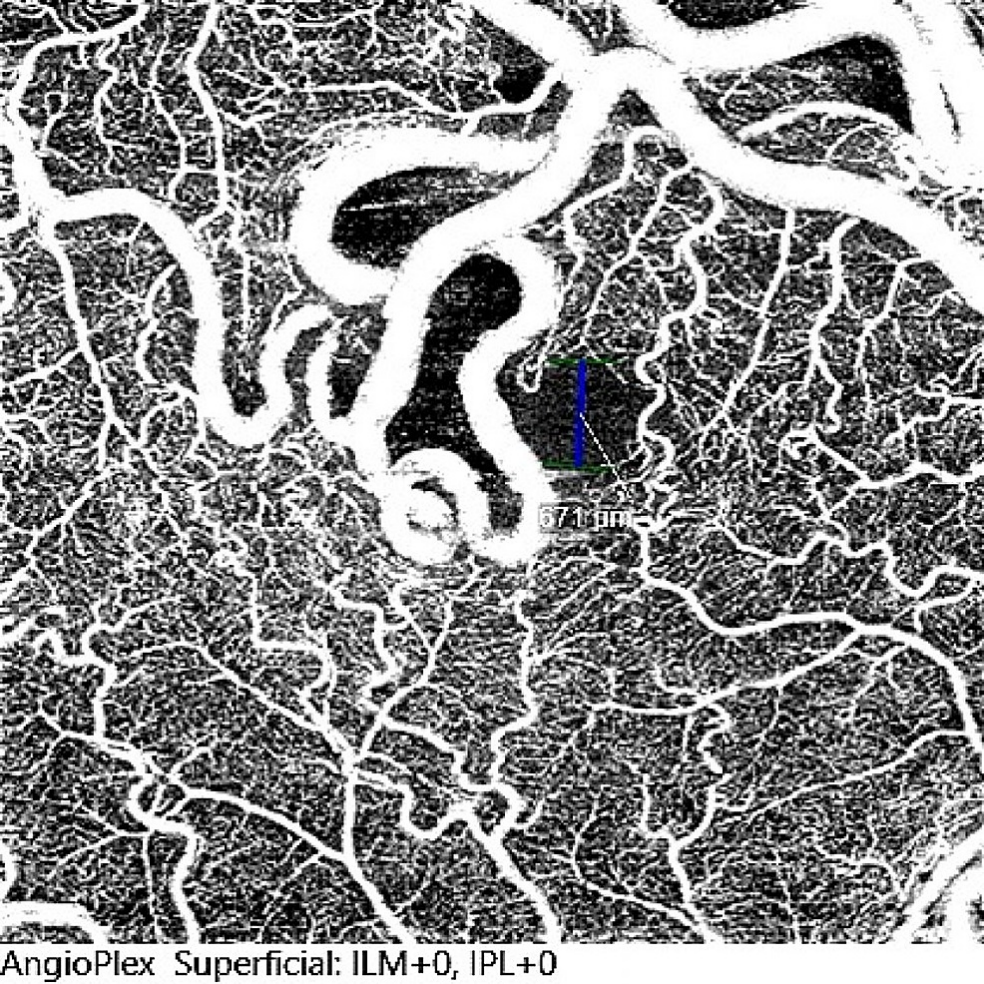
Superficial slab showing dilated and tortuous vessels in the right eye without a neovascular network.

**Figure 4:**
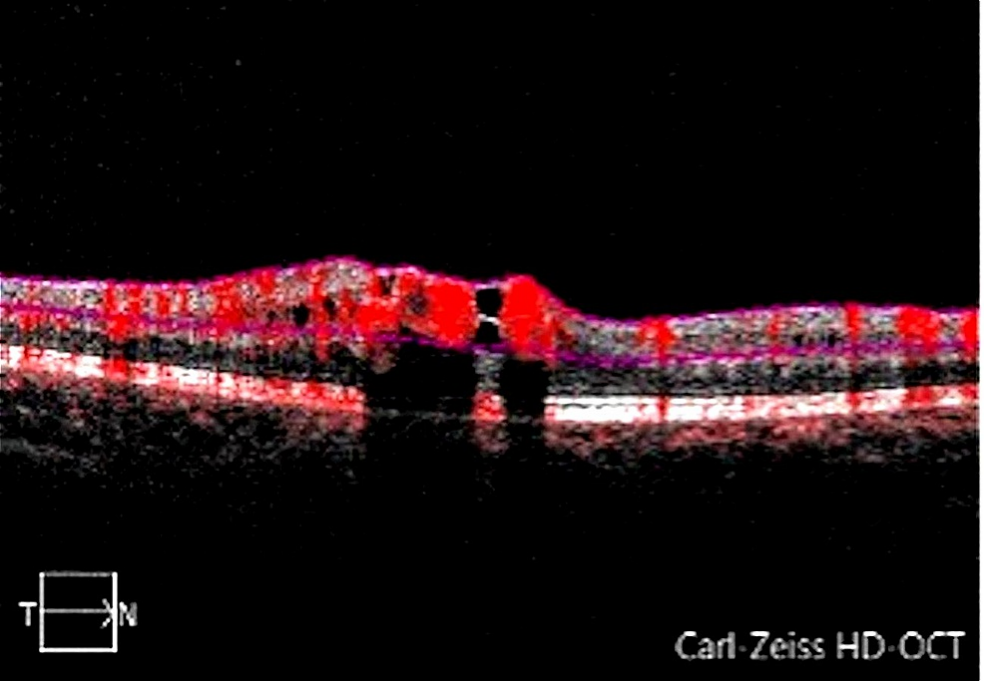
Corresponding OCT-A showing superficial retinal layers. OCT-A: optical coherence tomography angiography.

Spectral-domain optical coherence tomography (SD-OCT) examination using the SPECTRALIS® (Heidelberg Engineering, Heidelberg, Germany) showed distortion in the area of the enlarged vessels with minimal retinal fluid and prominent shadowing artefact (Figure 2). OCT-A of the macula using the AngioPlex® (ZEISS, Oberkochen, Germany) showed dilated and tortuous vessels only involving the superficial retinal layer without a neovascular network (Figures 3-4) 

**Figure 5:**
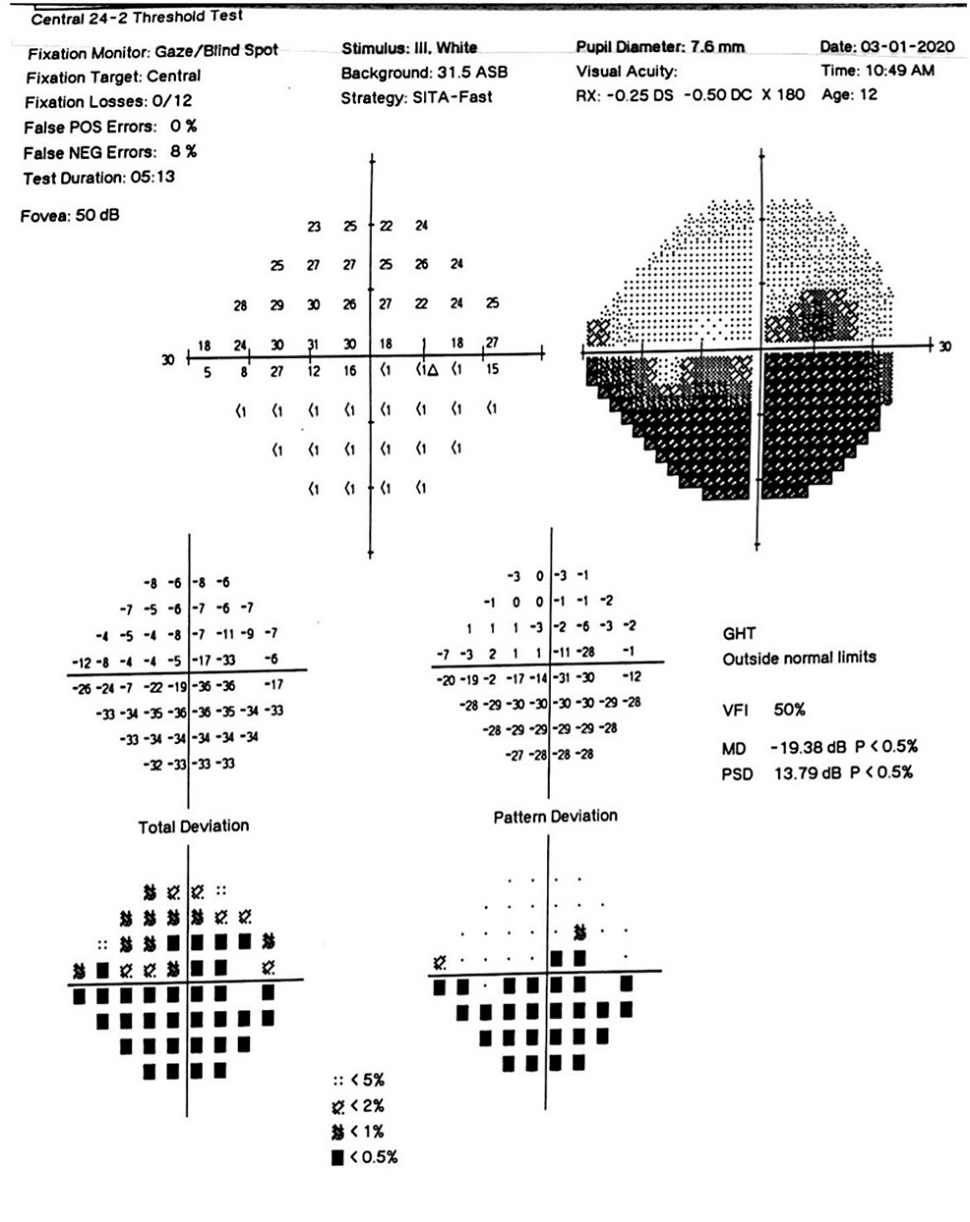
Humphrey visual field showing inferior field defect in the right eye.

Humphrey visual field assessed with the Humphrey Visual Field analyser (Carl Zeiss Meditec, Inc., Dublin, CA) of the right eye showed a defect in the inferior hemifield corresponding to the fundus findings (Figure 5). Fundus fluorescein and indocyanine angiography (FFA/ICG) were not done as the parents did not consent to the examination.

**Figure 6:**
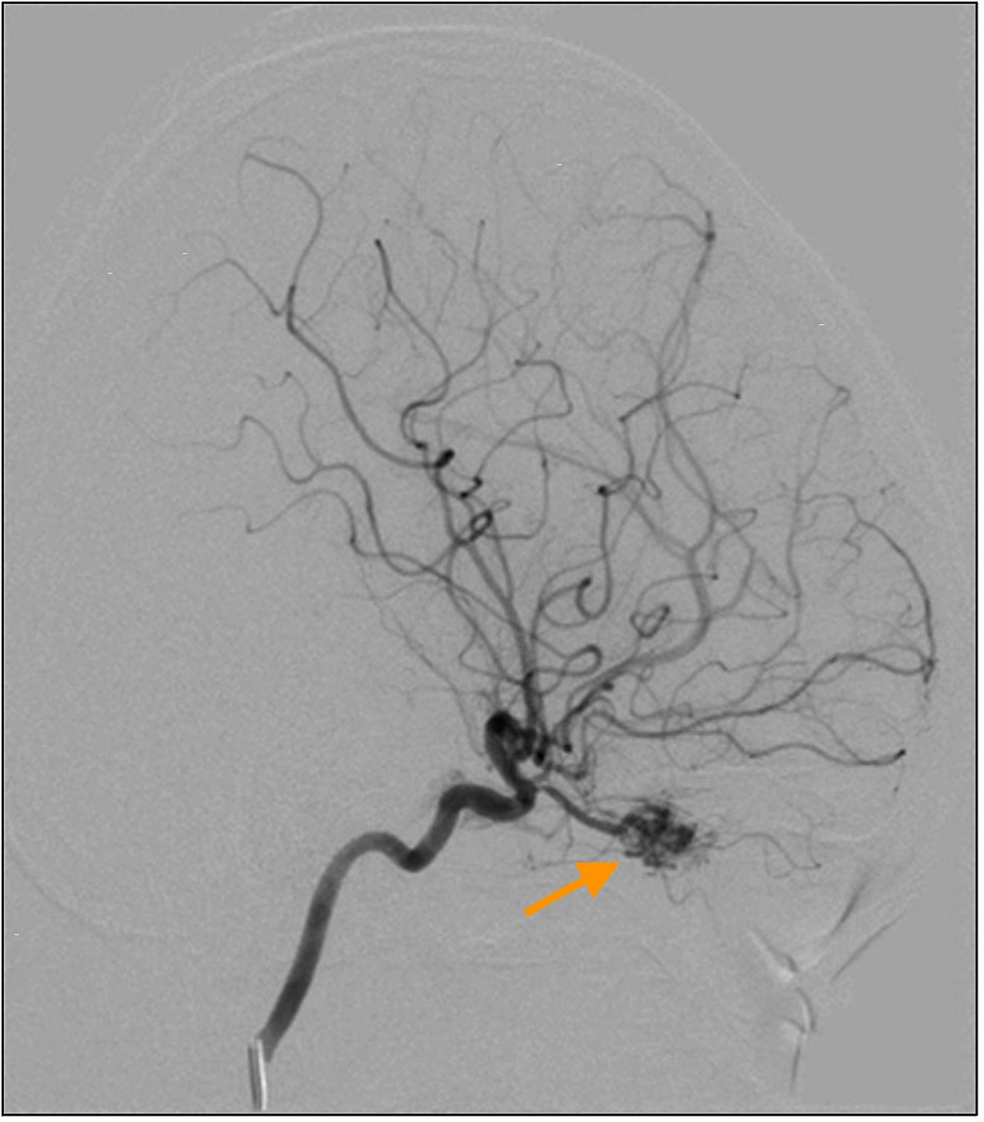
Right internal carotid artery angiogram lateral view showing the presence of an abnormal tangle of small vessels or nidus within the right orbit.

**Figure 7:**
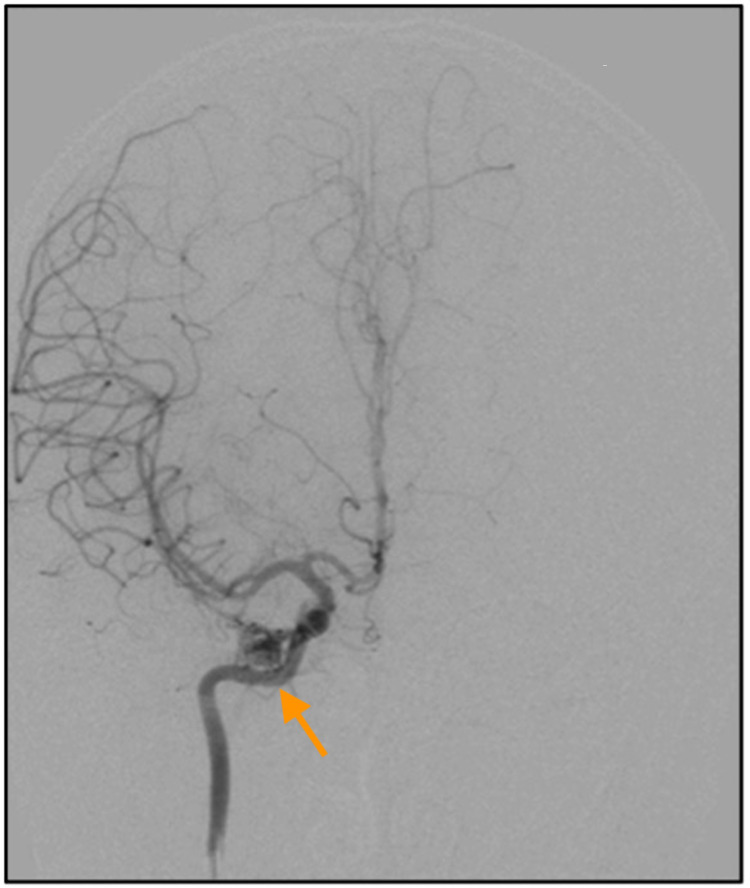
Right internal carotid artery antero-posterior view.

As there is a risk of central nervous system vascular malformation, magnetic resonance angiography/magnetic resonance venography (MRA/MRV) of the brain was performed. The right intra-orbital vascular channels surrounding the atrophied right optic nerve with gliosis, enlarged right ophthalmic artery, and opacification of the right inferior ophthalmic vein were seen which may suggest underlying dural fistula. A cerebral angiogram revealed the presence of an abnormal tangle of small vessels or nidus within the right orbit measuring 1.5 cm × 0.8 cm × 1.0 cm (AP×W×CC) which receives supply from a dilated right ophthalmic artery and early draining vein into the superior ophthalmic vein, which subsequently drains into the cavernous sinus, inferior petrosal sinus, and inferior jugular vein. Minimal reflux of contrast into the inferior ophthalmic vein was seen. This indicates the presence of right retroorbital AVM (Figures 6-7).

The patient was referred to the neurosurgery department. However, no intervention was done since embolization or resection of the dilated vessel might further impede her vision. The family opted for conservative management. During the last follow-up, there was no change in visual acuity and retinal changes. She did not have any new complaint; thus, the patient was scheduled for a routine follow-up visit after six months to monitor for any drop in visual acuity or development of macula oedema.

## Discussion

Racemose haemangioma is a rare congenital AVM condition that is not inherited. It has no gender predilection and there is no report on ethnic preference. It is due to mis-differentiations of the embryological vasculature during vasculogenesis [1].

Archer et al. classified racemose hemangioma into three groups. Group one is characterized by an abnormal capillary plexus between arterioles and venules that may not cause any clinical symptoms [1]. Group two is a direct arteriovenous communication without the interposition of capillary or arteriolar elements. The dilated vessels may superficially resemble those of a capillary hemangioma, but no tumor, exudation, or retinal detachment is present. Group three cases have severely dilated tortuous blood vessels often visible throughout the whole fundus area and most commonly associated with changes in the central nervous system, known as Wyburn-Mason syndrome. Based on our findings, our patient can be classified as Archer type three AVM. Magnetic resonance imaging excluded associated intracranial pathology (Wyburn-Mason syndrome). Retinal AVM is commonly observed in patients with a mean age of 25.6 years [2]. Only two cases of childhood RRH were ever reported [3,4]

Diminished vision in RRH is uncommon. It is rare for optic neuropathy to be observed in RRH and, to our knowledge, no case has been reported in a child. However, this is seen in our patient and may be due to the compressive effect of the enlarged AVM resulting in a loss of retinal nerve fibre layer and a decrease in the number of ganglion cells. Loss of ganglion cell bodies and axons in the affected retina has also been reported. The glial compartment is compressed and distorted by these aberrant vascular malformations [5]. This nerve fibre loss may explain the inferior visual field defects. Our patient may have significantly decreased vision because of optic nerve compression, retinal nerve fibre loss, and possible amblyopia. The usual visual disturbances seen in RRH are via various ocular complications such as venous occlusion, ocular ischemia, secondary glaucoma with rubeosis of the iris, and tractional retinal detachment [1,6-10].

Traditionally, FFA is the imaging of choice to visualize arteriovenous connections and any other retinal vascular malformation. The emergence of new imaging techniques, such as OCT-A, could also help in describing these vascular lesions. In RRH, dilated tortuous vessels were only visible in the more superficial retinal layers with the absence of any pigmented changes. These findings of RRH in OCT-A proved to be reproducible in all reported cases [3,11,12]. Hence, OCT-A may be an alternative imaging modality in exchange for FFA as it can define the depth of the lesion, is of high resolution, and permits easy follow-up of the lesions during treatment, without the risk, although rare, of anaphylaxis due to intravenous fluorescein used in FFA, especially when dealing with younger age group [13]. This non-invasive and painless imaging modality is preferred for children. However, in the presence of peripheral lesions, FFA is a better option than OCT-A since it is capable of identifying peripheral fundus lesions that could not be captured on OCT-A. FFA also provides information on the dynamic retinal circulation and lesion activity by revealing ongoing leakage. Nevertheless, OCT can also detect the presence of intraretinal or subretinal fluid and is useful for monitoring.

As intraorbital RRH are rare, their management poses a challenge for multidisciplinary teams. Arteriography is essential for confirming the diagnosis and identifying the arterial supply and venous drainage pathways [14,15]. Treatment for RRH includes embolization, sclerotherapy, surgical excision, or a combination of these within a multidisciplinary setting [16,17]. New anti-vascular endothelial growth factor (VEGF) agents have been tested for the treatment of malformed vessels or macular edema complicated with retinal vein occlusion [18,19]. The ultimate aim of treatment is the complete eradication of the lesion, reduction of symptoms, and improvement of function. However, the treatment may not be possible due to the location and the extent of the AVM [20]. The site of AVM in this patient is not suitable for any treatment since the embolization of the right ophthalmic artery could cause central retinal artery occlusion and further worsen her vision and the use of anti-VEGF is not indicated as no complication seen with this AVM.

## Conclusions

In conclusion, RRH may present in the paediatric age group, and optic nerve atrophy could be one of its complications. While both FFA and OCT-A have similar capabilities in characterizing retinal and vascular disorders, OCT-A may be the preferred investigation modality as it is non-invasive and painless for the patient. However, as the site of AVM was not amendable to surgery due to its location and severity, optic neuropathy was treated conservatively.
